# Preschool development, temperament and genetic liability as early markers of childhood ADHD: A cohort study

**DOI:** 10.1002/jcv2.12099

**Published:** 2022-09-02

**Authors:** Esther Tobarra‐Sanchez, Lucy Riglin, Sharifah S. Agha, Evie Stergiakouli, Anita Thapar, Kate Langley

**Affiliations:** ^1^ Child and Adolescent Psychiatry Section Division of Psychological Medicine and Clinical Neurosciences and MRC Centre for Neuropsychiatric Genetics and Genomics School of Medicine Cardiff University Cardiff UK; ^2^ Cwm Taf Morgannwg University Health Board Cardiff UK; ^3^ MRC Integrative Epidemiology Unit University of Bristol Bristol UK; ^4^ Population Health Sciences Bristol Medical School University of Bristol Bristol UK; ^5^ School of Psychology Cardiff University Cardiff UK

**Keywords:** ADHD, ALSPAC, developmental delay, early markers, polygenic risk scores, precursors, temperament

## Abstract

**Background:**

ADHD is associated with multiple adverse outcomes and early identification is important. The present study sets out to identify early markers and developmental characteristics during the first 30 months of life that are associated with ADHD 6 years later.

**Methods:**

9201 participants from the prospective Avon Longitudinal Study of Parents and Children (ALSPAC) birth cohort were included. Outcome measures were parent‐rated ADHD symptom scores (Strengths and Difficulties Questionnaire, SDQ) and ADHD diagnosis (Development and Wellbeing Assessment, DAWBA) at age 7. Seventeen putative markers were identified from previous literature and included: pre‐ and peri‐natal risk factors, genetic liability (ADHD polygenic risk scores, PRS), early development, temperament scores and regulatory problems. Associations were examined using regression analysis.

**Results:**

Univariable regression analysis showed that multiple early life factors were associated with future ADHD outcomes, even after controlling for sex and socio‐economic status. In a multivariable linear regression model; temperament activity scores (*B* = 0.107, CI = 0.083–0.132), vocabulary delay (*B* = 0.605, CI = 0.211–0.988), fine motor delay (*B* = 0.693, CI = 0.360–1.025) and ADHD PRS (*B* = 0.184, CI = 0.074–0.294) were associated with future symptoms (*R* ^2^ = 10.7%). In a multivariable logistic regression model, ADHD PRS (OR = 1.39, CI = 1.10–1.77) and temperament activity scores (OR = 1.09, CI = 1.04–1.16) showed association with ADHD diagnosis.

**Conclusion:**

As well as male sex and lower socio‐economic status, high temperament activity levels and motor and speech delays in the first 30 months of life, are associated with childhood ADHD. Intriguingly, given that genetic risk scores are known to explain little of the variance of ADHD outcomes, we found that ADHD PRS added useful predictive information. Future research needs to test whether predictive models incorporating aspects of early development and genetic risk scores are useful for predicting ADHD in clinical practice.

**Key points**

- Identifying early antecedents and markers of ADHD is important to support early detection and intervention.
- In a large comprehensive study of children from a population cohort, we identified that ADHD PRS, fine motor delays at 18 months, speech delays and higher temperament activity both at 24 months, showed independent association with ADHD traits at age 7. ADHD PRS and temperament activity were also associated with age 7 ADHD diagnosis.
- This study is the first to suggest that ADHD PRS, when combined with clinical and developmental variables, is associated with later ADHD symptoms and diagnosis.
- This study potentially helps inform practitioners which children may need more intensive surveillance or prevention strategies to support their later ADHD.
- Future research could investigate the specificity and potential causality of the identified markers and antecedents.

## INTRODUCTION

Attention‐deficit/hyperactivity disorder (ADHD) is a highly heritable neurodevelopmental disorder that typically onsets early in life (Thapar & Cooper, 2016). Attention‐deficit/hyperactivity disorder is associated with later educational failure, social and family difficulties, substance misuse, criminality, mental health disorders (Pelham et al., 2007), maltreatment and victimisation (Dinkler et al., 2017).

Although ADHD is often first identified from school‐age, epidemiological surveys suggest that rates in pre‐schoolers are similar to those in older children and adolescents (Egger & Angold, 2006). Also, a growing body of research suggests that the manifestation of core ADHD symptoms and diagnoses may be preceded by earlier risk markers, including infant development, temperament and neural markers (Johnson et al., 2015; Shephard et al., 2022). Identifying early risk markers is important as early detection and intervention could alter adverse developmental trajectories, especially during critical periods of child development, when the brain is rapidly developing and neuroplasticity is highest (Sonuga‐Barke et al., 2011).

A large body of research has focused on early temperament and infant behaviour as antecedents for ADHD (Nigg et al., 2004; Willoughby et al., 2017). Meta‐analyses and prospective cohorts have shown that aspects of temperament such as multiple and persistent regulatory problems during the first months of life (e.g., excessive crying, feeding, and sleeping difficulties), are also associated with later behavioural and ADHD outcomes (Baumann et al., 2019; Hemmi et al., 2011; Schmid & Wolke, 2014).

Studies investigating acquisition of early developmental milestones in children with ADHD indicate poorer motor skills and language development scores in children with, compared to without, ADHD (Arnett et al., 2013; Havmoeller et al., 2019; Lemcke et al., 2016, Sephard et al., 2022), although a recent review (Athanasiadou et al., 2020) suggests that differences in early motor development are non‐specific to ADHD.

Other early infant studies have focused on neonatal and postnatal measures of head circumference and there is some evidence to suggest that microcephaly at birth is associated with an increased risk of ADHD (Aagaard et al., 2018; Ferrer et al., 2019).

ADHD is multifactorial in origin, and early markers include known risk factors as well as infant antecedents. The most well‐established risk factors for ADHD include preterm birth, low birth weight and genetic liability (Sucksdorff et al., 2015; Thapar et al., 2013), others include restricted foetal growth (Murray et al., 2016), younger maternal age (Galéra et al., 2011) and advanced paternal age (D’Onofrio et al., 2014).

Genetic factors contribute to ADHD (Thapar & Cooper, 2016) with genome‐wide association studies (GWAS) suggesting that numerous common genetic variants contribute risk (Demontis et al., 2019). Polygenic risk scores (PRS) represent an individual's estimated total burden of common risk alleles for a particular disorder and ADHD PRS have been shown to be associated with ADHD across clinical and population samples (Stergiakouli et al., 2015).

To our knowledge, no longitudinal studies have undertaken a comprehensive examination of the simultaneous contribution of multiple infant early markers of ADHD and little research has successfully identified clinically informative early predictors. We used a UK population‐based birth cohort and sought to identify early markers during the first 30 months of life associated with ADHD symptom scores or DSM‐IV ADHD diagnosis. Both ADHD symptoms and diagnoses were explored to provide clinically relevant information, whilst also recognising that ADHD behaviours are dimensional, with similar risks associated across the continuum (Thapar & Cooper, 2016). Markers included pre‐perinatal factors (intra‐uterine growth restriction, maternal age at birth, preterm birth, APGAR score and head circumference at birth), early developmental difficulties (including speech and motor delays), infant temperament dimensions of activity and distractibility, regulatory problems (early sleeping, crying, and feeding problems), and ADHD PRS.

## METHODS

### Sample

We analysed data from the Avon Longitudinal Study of Parents and Children (ALSPAC), a prospective longitudinal population‐based birth cohort of a representative sample of parents and children (Boyd et al., 2013; Fraser et al., 2013) Our sample included a total of 9201 children with information on ADHD measures at age 7 years. Where families included multiple births (twins), we included only the oldest sibling. Specific details of the sample are provided in the Supporting Information.

## MEASURES

### Ratings of ADHD at age 7 years

#### Strengths and Difficulties Questionnaire (SDQ)

The SDQ is a brief, extensively evaluated behavioural screening questionnaire for children aged 3–16 years, with demonstrated reliability and validity (Goodman, 2001) and was used to provide a continuous, symptom score at age 7 years. The five questions in the hyperactivity/inattention subscale rated on a 3‐point scale are summed to give a total score. Data were available for 8302 children.

#### Development and Wellbeing Assessment (DAWBA)

The DAWBA structured assessment was sent as a paper questionnaire to carers and teachers when the children were 7 years old and provided information for ADHD diagnoses. The ADHD section assesses inattention, hyperactivity, impulsivity, age of onset and impairment. In accordance with the DAWBA protocol, initially the DAWBA computer algorithm ascertained the probability of having ADHD for each participant (Goodman et al., 2011; www.dawba.info). Child psychiatrists then reviewed the data for each participant, to determine a final definitive DSM‐IV diagnosis of ADHD.

DAWBA data were available for 8121 children. Good validity of the diagnoses assigned with the DAWBA has been confirmed (Goodman et al., 2000).

### Potential early markers

#### Genetic data

We used previously calculated PRSs for ADHD, defined using alleles associated at *p* < .05 in an independent GWAS (Demontis et al., 2019). Details of how the ADHD PRS scores were derived are provided in the Supporting Information. Scores were standardised using Z‐score transformation.

#### Parental age

These variables were dichotomised to make them clinically relevant. Younger maternal age was defined as less than 20 years and advanced paternal age as above 45 years (D’Onofrio et al., 2014; Galéra et al., 2011).

#### Perinatal factors

Intra‐uterine Growth Restriction (IUGR), preterm birth and APGAR score at 5 min were obtained from obstetric clinical records. Head circumference (HC) at birth was assessed by trained ALSPAC staff.

Preterm birth was defined as those born at <37 weeks of gestation based on best clinical estimate of expected date of delivery. APGAR score is a measure of vitality of a new‐born infant, routinely calculated by the attending clinician after every birth (Cnattingius et al., 2017), and IUGR is a clinical definition implying a pathological restriction of the growth potential in utero, recorded as present or absent. APGAR score at 5 min after birth was dichotomised to define healthy new‐borns (score of 10) and those with some problems after birth (score of 0–9). Individuals with a head circumference ≤2 SD below the mean for their sex were considered to have microcephaly.

#### Concerns over vision and hearing

At age 15 months, parents were asked as part of a paper questionnaire if their child had been referred to a Hearing Assessment Centre or Eye Specialist. Where the answer was “yes” for either question children were considered to have early concerns with vision or hearing.

#### Motor development

Fine and gross motor development were assessed at 18 months old using an adapted version of the Denver Developmental Screening Test (DDST; Frankenburg & Dodds, 1967). Parents completed a questionnaire about their child demonstrating 16 fine motor and 12 gross motor skills rated on a three‐point scale (0 = not yet started, 1 = once or twice, 2 = yes, can do well). Analysed motor scores were age adjusted z‐scores. To identify a clinically relevant category of motor developmental delay, a dichotomous variable was created for each score with motor skills >1 SD z‐score below the mean, indicating the presence or absence of motor delays.

#### Speech development

Vocabulary and grammar skills were assessed at 24 months old with the ALSPAC adaptation of the MacArthur‐Bates Communicative Development Inventory (MB‐CDI; Feldman et al., 2000). The Vocabulary section of the MB‐CDI contains a checklist of 123 words. Parents rated their child's use of each word on a three‐point scale (2 = child says, 1 = child understands, 0 = neither). Answers were summed to give a Vocabulary score. The Grammar section contains 29 items with questions about basic use of grammar (0 = not yet, 1 = sometimes, 2 = often), use of plurals and past tense (2 = says, 1 = understands, 0 = neither), summed to give a Grammar Score. Vocabulary and grammar scores were dichotomised as well with scores >1SD below the mean, to create the clinically relevant categories of vocabulary and grammar delays.

#### Temperament

Mothers completed an 89‐item adapted version of the Carey Temperament Scale (CTS; Fullard et al., 1984) with questions about frequency of child behaviour at age 2 years rated on a five‐point scale (1 = almost never, 2 = rarely, 3 = sometimes, 4 = often, 5 = almost always). 10 Distractibility items (effectiveness of extraneous stimuli in altering the direction of ongoing behaviour) and nine Activity items (level of the motor component in the child's functioning) were summed to give the relevant sub‐scores.

#### Regulatory problems

Parents completed questionnaires about their child's feeding (at age 24 months), sleeping and crying (at 30 months) habits and these questions were utilised to assess regulatory problems, following a previously used scoring system. The presence of seven sleeping problems and six feeding problems were assessed on a 4‐point scale used to identify the presence of a problem (1–3 vs. 0) and summed to create total scores. A total crying problem score was derived by summing the answers to five items and binary clinically relevant crying, sleeping and feeding variables were defined as >1 SD above the sample mean, in‐line with previous work (Winsper & Wolke, 2014).

Details of how scores were derived prorated measures were used for SDQ Score, Denver motor score, Carey temperament score and regulatory problems score where <50% items were missing.

#### Covariates

Sex and socioeconomic status (SES) were included as covariates because ADHD is more common in males and in more socially disadvantaged groups (Thapar & Cooper, 2016). SES was based on mother's occupation during pregnancy coded using the Standard Occupational Classification SOC (OPCS, 1991) and dichotomised in‐line with previous work (Eyre et al., 2019; manual and non‐manual skilled occupations, partly skilled occupations and unskilled occupations vs. professional occupations and managerial and technical occupations).

### Statistical analyses

Analyses were undertaken using SPSS version 21. Linear and logistic regression analyses were conducted to investigate associations between 17 early markers and ADHD symptoms (assessed using the SDQ) and diagnosis (assessed using the DAWBA), respectively. First, a univariable model analysed each predictor separately (Model 1), then adjusted for sex (Model 2) and SES (Model 3). Variables showing an association in the univariable adjusted model (Model 3), were subsequently included in a fully adjusted multivariable model. We used Bonferroni correction (*α* = 0.05/number of significant tests) in Model 3, prior to conducting multivariate regression analysis, to correct for multiple testing. Sensitivity analyses, were conducted separately for females and males to evaluate if predictors varied by sex (Tables S2 and S3).

## RESULTS

We analysed data from 9201 children (51.3% males). Descriptive statistics for all study variables can be found in Table 1.

**TABLE 1:** Descriptive statistics for all study variables

	*N*	Mean (95% CI)/%(N)
**Outcome measures**		
*SDQ score*	8302	3.39 (*SD* = 2.36)
Boys	4261	3.78 (*SD* = 2.45)
Girls	4041	2.97 (*SD* = 2.20), *t*(8275) = 15.9, *p* < .001
*DAWBA ADHD diagnosis*	8121	Yes = 2.1% (*N* = 174)
Boys		3.5% (*N* = 147)
Girls		0.7% (*N* = 27), χ^2^(1) = 78,8, *p* = <.001
**Demographic factors**		
*Sex*	9201	Males = 51.3% (4724)
*Maternal age at birth*	8709	28.89 (28.79–28.99)
<20 years		3.7%
*Paternal age at birth*	7571	30.02 (29.88–30.17)
>45 years		2.1% (161)
**Perinatal factors**		
*Preterm birth*	5578	7.3% (406)
*APGAR < 10*	5388	41.6% (2244)
*IUGR*	5617	2.8% (160)
*Head circumference*	5934	34.86 (34.83–34.90)
>2 SD below mean		2.3% (134)
**Early developmental concerns**		
*Eye/hearing concerns*	8567	4.8% (415)
*Gross motor*	8382	−0.0062
>1SD below mean (gross motor delay)		11.4% (959)
*Fine motor*	8347	0.0189 (−0.002–0.039)
>1SD below mean (fine motor delay)		14.6% (1219)
*Vocabulary*	8461	152.5 (151.3–153.7)
>1SD below mean (vocabulary delay)		18% (1527)
*Grammar*	7878	19.69 (19.36–20.02)
>1SD below mean (grammar delay)		15.2% (1195)
**Temperament**		
*Activity*	8413	23.14 (23.04–23.24)
*Distractibility*	8411	24.61 (24.51–24.71)
**Regulatory problems**		
*Feeding difficulties*	8460	2.84 (2.79–2.87)
>1SD above mean		19.8% (1674)
*Sleeping problems*	8305	3.09 (3.04–3.00)
>1SD above mean		14.1% (1172)
*Excessive crying*	8382	0.11 (0.10–0.12)
>1SD above mean		6.7% (561)

There was no evidence of multicollinearity between early markers (see Table S1), therefore all were included in regression models (Ernst & Albers, 2017).

### Associations between early markers and ADHD symptom scores

Seven early markers showed association with ADHD scores in univariable analysis when controlling for sex and SES (Table 2, Model 3): ADHD PRS, fine motor delays, vocabulary delays, grammar delays, higher temperament activity, higher temperament distractibility and feeding difficulties (Bonferroni correction threshold *p* < .004). There was also weaker evidence of associations with younger mothers at birth, IUGR and microcephaly at birth.

**TABLE 2:** Associations between ADHD symptoms and early markers and precursors

Predictors	Model 1 (unadjusted)				Model 2 (adjusted by sex)				Model 3 (adjusted by sex and SES)				Model 4, Multivariable (adjusted), *N* = 3777, *R* ^2^ = .107		
	*N*	Unstandard.B (95% CI)	Stand.B	*P*	*N*	Unstandard.B (95% CI)	Stand.B	*P*	*N*	Unstandard.B (95% CI)	Stand.B	*P*	Unstandard. B (95% CI)	Stand. B	*P*
ADHD PRS	5509	0.229 (0.167, 0.291)	0.097	<.001	5509	0.230 (0.169, 0.290)	0.098	<.001	4719	0.198 (0.132, 0.264)	0.084	<.001	0.167 (0.097, 0.238)	0.072	<.001
Mother <20 years at birth	7976	0.627 (0.342, 0.913)	0.048	<.001	7976	0.633 (0.351, 0.914)	0.049	<.001	6703	0.392 (0.049, 0.735)	0.027	.025	‐	‐	‐
Father >45 years at birth	6954	0.178 (−0.212, 0.568)	0.011	.371	6954	0.141 (−0.243, 0.525)	0.009	.471	6028	0.087 (−0.32, 0.49)	0.005	.674	‐	‐	‐
Prematurity	5047	0.302 (0.043, 0.56)	0.032	.022	5047	0.243 (−0.012, 0.497)	0.026	.062	4230	0.252 (−0.03, 0.53)	0.026	.081	‐	‐	‐
IUGR	5082	0.475 (0.07, 0.881)	0.032	.022	5082	0.497 (0.098, 0.895)	0.034	.015	4260	0.573 (0.140, 1.006)	0.039	.009	‐	‐	‐
Microcephaly	5403	0.496 (0.076, 0.916)	0.031	.021	5403	0.493 (0.078, 0.908)	0.031	.020	4498	0.408 (−0.062, 0.878)	0.025	.089	‐	‐	‐
APGAR	4874	0.121 (−0.015, 0.257)	0.025	.081	4874	0.118 (−0.016, 0.252)	0.024	.085	4077	0.098 (−0.047, 0.244)	0.020	.186	‐	‐	‐
Hear/Vision referral	7854	0.179 (−0.06, 0.422)	0.016	.149	7854	0.156 (−0.084, 0.396)	0.014	.203	6609	0.154 (−0.11, 0.419)	0.014	.253	‐	‐	‐
Fine motor delay	7720	0.820 (0.675, 0.965)	0.126	<.001	7720	0.769 (0.626, 0.911)	0.118	<.001	6500	0.724 (0.564, 0.884)	0.108	<.001	0.521 (0.309, 0.734)	0.075	<.001
Gross motor delay	7755	0.047 (−0.116, 0.211)	0.006	.57	7755	0.078 (−0.083, 0.239)	0.011	.343	6527	0.117 (−0.059, 0.292)	0.016	.193	‐	‐	‐
Vocabulary delay	7832	0.832 (0.697, 0.968)	0.135	<.001	7832	0.679 (0.543, 0.815)	0.110	<.001	6576	0.593 (0.443, 0.744)	0.094	<.001	0.349 (0.098, 0.600)	0.084	.007
Grammar delay	7306	0.473 (0.324, 0.621)	0.073	<.001	7306	0.355 (0.207, 0.502)	0.054	<.001	6148	0.338 (0.178, 0.498)	0.052	<.001	0.081 (−0.145, 0.306)	0.012	.484
Activity	7791	0.138 (0.127, 0.149)	0.266	<.001	7791	0.131 (0.120, 0.142)	0.252	<.001	6549	0.134 (0.122, 0.146)	0.259	<.001	0.123 (0.107, 0.139)	0.240	<.001
Distractibility	7790	0.020 (0.009, 0.031)	0.039	.001	7790	0.022 (0.011, 0.033)	0.044	<.001	6549	0.028 (0.016, 0.039)	0.055	<.001	0.005 (−0.010, 0.020)	0.010	.535
Sleeping >1SD	7526	0.074 (−0.079, 0.227)	0.011	.346	7526	0.080 (−0.071, 0.230)	0.012	.31	6276	0.072 (−0.09, 0.235)	0.011	.388	‐	‐	‐
Crying >1SD	7591	0.210 (−0.003, 0.423)	0.022	.053	7591	0.228 (0.018, 0.439)	0.024	.033	6327	0.124 (−0.108, 0.356)	0.013	.293	‐	‐	‐
Feeding >1SD	7668	0.212 (0.079, 0.345)	0.036	.002	7668	0.198 (0.067, 0.329)	0.033	.003	6390	0.207 (0.064, 0.350)	0.035	.004	0.110 (−0.067, 0.287)	0.019	.222

Abbreviations: ADHD, Attention Deficit and Hyperactivity Disorder; IUGR: Intrauterine Growth Restriction; PRS, Polygenic Risk Scores.

In multivariable adjusted analysis (Model 4), four markers remained associated with ADHD scores: ADHD PRS, fine motor delay, vocabulary delay and higher temperament activity (see Table 2).

Stratified multivariable analysis showed that associations were generally consistent across males and females, except that associations with vocabulary delays were only seen in males (See Table S2).

### Associations between early markers and ADHD diagnosis

When controlling for sex and SES, ADHD diagnosis was associated with five early markers (Table 3, Model 3): ADHD PRS, fine motor delay, high temperament activity, vocabulary delay and grammar delay (Bonferroni correction threshold *p* < .006). There was also weaker evidence of association with gross motor delay. In the multivariable model (Model 4), ADHD PRS and temperamental activity showed strong evidence of association (see Table 3). Due to the limited number of girls with ADHD diagnoses (*N* = 27), stratified analysis was carried out only for males which were generally consistent with those in the whole sample (Table S3).

**TABLE 3:** Associations between ADHD diagnosis and early markers and precursors

Predictors	Model 1			Model 2 (adjusted by sex)			Model 3 (adjusted by sex and SES)			Model 4, multivariable (adjusted sex & SES) *N* = 3978	
	*N*	OR (95% CI)	*P*	*N*	OR (95% CI)	*P*	*N*	OR (95% CI)	*P*	OR (95% CI)	*P*
ADHD PRS	5491	1.35 (1.12, 1.62)	.001	5491	1.36 (1.13, 1.64)	.001	4673	1.38 (1.12, 1.70)	.002	1.39 (1.09, 1.77)	.006
Mother <20 years at birth	7731	2.36 (1.29, 4.32)	.005	7731	2.41 (1.31, 4.43)	.005	6515	2.05 (0.97, 4.32)	.057	‐	‐
Father >45 years at birth	6771	0.69 (0.25, 1.9)	.477	6771	0.66 (0.24, 1.82)	.424	5890	0.55 (0.19, 1.54)	.257	‐	‐
Prematurity	4910	1.82 (1.04, 3.15)	.033	4910	1.66 (0.95, 2.9)	.071	4116	1.77 (0.97, 3.22)	.062	‐	‐
IUGR	4949	1.72 (0.74, 3.98)	.203	4949	1.81 (0.78, 4.23)	.166	4149	1.74 (0.69, 4.41)	.240	‐	‐
Microcephaly	5241	0.41 (0.06, 2.99)	.383	5241	0.392 (0.05, 2.84)	.354	4364	0.51 (0.07, 3.73)	.507	‐	‐
APGAR	4749	1.08 (0.759, 1.56)	.642	4749	1.09 (0.76, 1.57)	.615	3972	1.11 (0.74, 1.65)	.606	‐	‐
Hear/Vision referral	7621	2.16 (1.25, 3.72)	.005	7621	2.15 (1.24, 3.71)	.006	6427	1.79 (0.92, 3.48)	.084	‐	‐
Fine motor delay	7450	1.89 (1.29, 2.77)	.001	7450	1.77 (1.20, 2.59)	.003	6289	1.84 (1.21, 2.79)	.004	1.36 (0.75, 2.48)	.305
Gross motor delay	7476	1.54 (0.99, 2.38)	.052	7476	1.63 (1.05, 2.53)	.029	6312	1.78 (1.11, 2.87)	.016	‐	‐
Vocabulary delay	7551	2.66 (1.9, 3.7)	<.001	7551	2.66 (1.90, 3.71)	<.001	6354	2.19 (1.50, 3.18)	<.001	1.72 (0.91, 3.24)	.094
Grammar delay	7041	2.23 (1.5, 3.2)	<.001	7041	1.84 (1.26, 2.69)	.002	5940	1.88 (1.24, 2.85)	.003	1.49 (0.81, 2.74)	.200
Activity	7511	1.12 (1.08, 1.16)	<.001	7511	1.11 (1.07, 1.15)	<.001	6331	1.12 (1.07, 1.17)	<.001	1.09 (1.04, 1.16)	.001
Distractibility	7508	1.01 (0.98, 1.05)	.405	7508	1.01 (0.97, 1.04)	.541	6330	0.98 (0.94, 1.02)	.394	‐	‐
Sleeping >1SD	7346	1.22 (0.79, 1.86)	.366	7346	1.21 (0.78, 1.85)	.387	6114	1.23 (0.76, 1.98)	.392	‐	‐
Crying >1SD	7412	1.35 (0.77, 2.36)	.289	7412	1.42 (0.81, 2.48)	.219	6167	1.37 (0.71, 2.65)	.350	‐	‐
Feeding >1SD	7487	1.03 (0.69, 1.54)	.866	7487	1.01 (0.68, 1.51)	.934	6224	0.81 (0.50, 1.30)	.387	‐	‐

Abbreviations: ADHD, Attention Deficit and Hyperactivity Disorder; IUGR: Intrauterine Growth Restriction; PRS: Polygenic Risk Scores.

## DISCUSSION

In this prospective cohort, we observed that ADHD PRS, fine motor delays at 18 months, speech delays and higher temperament activity at 24 months showed independent association with ADHD traits at age seven. ADHD PRS and temperament activity were also associated with subsequent ADHD diagnosis.

The aspects of temperament and regulation analysed here are related to the core features of ADHD and could represent the starting point of a developmental trajectory of dysregulation (Baumann et al., 2019; Schmid & Wolke, 2014). Prospective studies and meta‐analysis (Kostyrka‐Allchorne et al., 2020; Willoughby et al., 2017) found modest associations with later psychopathology, and activity domains were particularly associated with ADHD and externalising disorders. Representing aspects of temperament, infant regulatory problems result in frequent help‐seeking and are easy to identify. Although not observed in this study, previous meta‐analysis showed that regulatory problems had low‐to‐medium effect sizes in predicting ADHD problems, although higher associations were found for excessive crying and highest effect sizes for sleeping problems (Hemmi et al., 2011).

The developmental antecedents found here are in line with those observed in other cohorts such as the national birth cohort in Denmark (Lemcke et al., 2016), in which observations at 18 months of age associated with ADHD were the child not being able to fetch things on request, having speech delays or being significantly more active than average.

The results of our study also suggest that genetic liability indexed as ADHD PRS were associated with subsequent ADHD diagnosis. Many studies have shown that participants with higher ADHD PRS have an increased chance of meeting ADHD diagnostic criteria, higher ADHD symptom levels and ADHD persistence (Riglin et al., 2016; Thapar, 2020). Although PRS are not strong predictors, when combined with clinical predictors they may have clinical utility in risk assessments both in Psychiatry and Child Health as well as for physical health conditions such as cardiovascular disease or cancer (Lewis & Vassos, 2020; Murray et al., 2021).

This study is the first to our knowledge to suggest that ADHD PRS, when combined with clinical and developmental variables, is associated with later ADHD symptoms and diagnosis. Our results may therefore have important practical implications because identifying precursors and antecedents of ADHD, even when not causal, could be important in risk assessments of ADHD, for example, determining follow‐up options for individuals who are at clinical risk of future illness. However, the clinical utility of these results needs evaluation especially as the variance explained by this group of early markers was limited.

As we learn more about the early manifestations of clinical psychopathology, we will also be able to suggest and evaluate preventive intervention approaches targeting some of the early predictors. Moreover, this area of research is particularly relevant for ADHD because, in contrast to Autism spectrum disorder, clinicians lack effective screening tools that help them to distinguish ADHD precursors (Carter et al., 2004).

### Methodological strengths and limitations

This study has several strengths, such as a large representative sample and prospectively collected information using well‐validated instruments. Although some variables are based on objective measures or obstetric data, most of the variables were collected through parental report, which may have introduced a measurement error. However, our variables represent data available in the real world and in general, evidence suggests that parent report and directly assessed measures of language and motor skills are significantly correlated (Miller et al., 2017; Torrens & Ruiz, 2021). We looked at dichotomised variables to indicate delay in development which may have decreased the statistical power to detect associations. However, this approach is more in line with clinical practise, whilst results were the same when looking at continuous scores. Furthermore, an adapted version of the Denver developmental scale was used, necessitating dichotomisation of the sample using sample‐specific rather than standardised scores.

The study also has some limitations. The ALSPAC cohort shows attrition (Taylor et al., 2018) necessitating replication. However, we found no differences in rates of ADHD symptoms and diagnosis in those with complete and some missing data (see Table S4 and Figure S1) and although selective attrition can lead to a loss of power and underestimation of the prevalence of psychiatric disorders, it has been shown in the ALSPAC sample that this is less likely to affect the patterns of associations between disorders and risk factors (Wolke et al., 2009). The prevalence of a diagnosis of ADHD was not high in this sample (2.1%) which may have limited our power to find associations with potential precursors. However, this prevalence rate is in line with other diagnostic prevalence rates in UK epidemiological studies using the DAWBA (e.g. NHS Digital, n.d.).

Also, ADHD shows a high level of co‐occurrence with other neurodevelopmental disorders (Thapar & Cooper, 2016) and it is not clear whether the developmental markers we identify are specifically associated with ADHD.

Future research may focus on interaction between predictors, non‐linear interactions and the specificity of early markers. It is also of interest to analyse the persistence of predictors in ADHD diagnosis at an older age or studying if early features represent markers or predictors at the individual level. Future research may also look at the use of the identified early markers and precursors to develop a prediction model for later ADHD. However, the use of prediction models within child and adolescent psychiatry has been shown to have limited utility, in part due to methodological issues such as sample size and replicability, which would have been seen in this study had we developed such a model (Larsson, 2021; Senior et al., 2021).

## CONCLUSION

The results of this study suggest that genetic liability indexed by ADHD PRS, aspects of temperament, and early developmental delays act as antecedents and represent early precursors of later ADHD.

This area of research has important clinical implications because it may inform practitioners about which children need more intense surveillance or preventive strategies. The combination of PRS with clinical and developmental variables has potential to contribute to risk assessment and aid clinical decision‐making.

## AUTHOR CONTRIBUTIONS

**Esther Tobarra‐Sanchez**: Conceptualization, Data Curation, Formal Analysis, Funding Acquisition, Methodology, Investigation, Visualization, Writing – Original Draft. **Lucy Riglin**: Resources, Writing Review and Editing. **Sharifah S. Agha**: Writing Review and Editing, Supervision. **Evie Stergiakouli**: Conceptualization, Funding Acquisition, Methodology, Supervision, Writing Review and Editing. **Kate Langley**: Conceptualization, Funding Acquisition, Formal Analysis, Investigation, Methodology, Project Administration, Supervision, Writing Review and Editing. **Anita Thapar**: Conceptualization, Funding Acquisition, Investigation, Methodology, Project Administration, Supervision, Writing Review and Editing.

## CONFLICT OF INTEREST

The authors declares that there is no conflict of interest that could be perceived as prejudicing the impartiality of the research reported.

## ETHICAL CONSIDERATIONS

Approval obtained from the ALSPAC Ethics and Law Committee.

## Supporting information

Supporting information S1

## Data Availability

Data sharing is not applicable to this article as no new data were created or analysed in this study.
