# Supplementary material for: Preschool development, temperament and genetic liability as early markers of childhood ADHD: A cohort study
Source: JCPP Adv. 2022 Sep 2;2(3):e12099. doi: 10.1002/jcv2.12099 (PMC9716640; doi:10.1002/jcv2.12099)
Supplement: Supplementary file 1 — Supporting information S1 [file JCV2-2-e12099-s001.docx]

**Supporting Information**

**Sample**

Pregnant women resident in Avon, UK with expected dates of delivery 1st April 1991 to 31st December 1992 were invited to take part in the study. The initial number of pregnancies enrolled is 14,541 (for these at least one questionnaire has been returned or a “Children in Focus” clinic had been attended by 19/07/99). Of these initial pregnancies, there was a total of 14,676 foetuses, resulting in 14,062 live births and 13,988 children who were alive at 1 year of age. Where families included multiple births, we included the oldest sibling.

When the oldest children were approximately 7 years of age, an attempt was made to bolster the initial sample with eligible cases who had failed to join the study originally. As a result, the total sample size for data collected after the age of seven is therefore 15,454 pregnancies, resulting in 15,589 foetuses. Of these 14,901 were alive at 1 year of age. Part of this data was collected using REDCap (<https://projectredcap.org/resources/citations/>). Participants were assessed at multiple time points since recruitment using questionnaire and clinic-based measures. Please note that the study website contains details of all the data that is available through a fully searchable data dictionary and variable search tool: http://www.bristol.ac.uk/alspac/researchers/our-data/. Further details of the study, measures and sample can be found elsewhere (Boyd et al., 2013; Fraser et al., 2013; Northstone et al., 2019).

Ethical approval for the study was obtained from the ALSPAC Ethics and Law Committee and the Local Research Ethics Committees. Consent for biological samples has been collected in accordance with the Human Tissue Act (2004) and informed consent for the use of data collected via questionnaires and clinics was obtained from participants following the recommendations of the ALSPAC Ethics and Law Committee at the time.

**Generating polygenic risk scores**

In total 9912 ALSPAC children were genotyped using the Illumina HumanHap500-quad genotyping array. Individuals were excluded on the basis of gender mismatches; minimal or excessive heterozygosity, disproportionate levels of individual missingness (>3%), insufficient sample replication (IBD <0.8), non-European ancestry (assessed by multidimensional scaling analysis and compared with Hapmap II) and cryptic relatedness (IBD > 0.1). SNPs were excluded based on minor allele frequency (<1%), call rate (<95%) or evidence for violations of Hardy-Weinberg equilibrium (P < 5E-7). Imputation was conducted by the ALSPAC team using Impute V2.2.2 against the 1000 genomes reference panel (Phase 1, Version 3: all polymorphic SNPs excluding singletons), using all 2186 reference haplotypes (including non-Europeans). SNPs were subsequently filtered based on minor allele frequency (<1%) and imputation quality (INFO<0.8). Following quality control and limiting individuals to one child per family, genetic data were available for N=7975.

Genome-wide association study (GWAS) were filtered to remove SNPs that were palindromic, insertions/deletions, non-autosomal, INFO score <0.8, missing in N>1 study and duplicates (<https://github.com/ricanney>).

PRS were generated for individuals in ALSPAC as the number of disorder risk alleles – defined using the GWAS summary statistics - weighted by effect size, using PRSice (Euesden et al., 2015); SNPs were clumped with an R^2^ threshold of 0.1 and a distance threshold of 1000kb and excluding the extended major histocompatibility complex (MHC; chromosome 6: 26-33Mb) due to the high linkage disequilibrium (LD) within this region. In the primary analyses we defined risk alleles as those associated with case-status at p<0.05 as this threshold has previously been shown to maximally capture phenotypic variance for schizophrenia (Schizophrenia Working Group of the Psychiatric Genomics Consortium, 2014). Polygenic risk scores were standardized using Z-score transformation.

**Regulatory problems score**

Six questions about the child’s feeding habits and seven questions about sleeping were included in questionnaires sent to parents at 24 months and 30 months of child age, respectively. Answers were assessed using a Likert scale (“0=No”, “1=yes, but not worried”, “2=yes, bit worried”, “3=yes, very worried”).

Five questions about frequency of crying were asked to parents when the child was 30 months old. Scoring for each item was as follows: “time spent crying compared to others” (more=1, same/less=0), “frequency of child fussing and whining” (long time every day=1, short time every day/several times per week/sometimes/rarely=0), “frequency child cries for no reason” (often=1, sometimes/rarely/never=0), “mother can calm child when crying” (never/with effort=1, after some time/easily/child never cries=0), “mother feels child’s crying is a problem” (yes=1, no=0).

To create total scores we followed a scoring system previously used (Winsper & Wolke, 2014). Responses about feeding and sleeping were recoded as the presence of a problem (scores 1, 2 and 3) or no problem (scores of 0) and were summed to create total scores. A total crying problem score was derived by summing the answers to the five items.

Prorating measures were calculated using mean imputation when the answer to only one question was missing for each score. As Winsper and Wolke did, we also created clinically relevant crying, sleeping and feeding variables with cut-off points of 1 SD above the sample mean.

**SDQ questionnaire, Denver Test and Carey scale prorated scores**

Prorated variables were generated using a mean proration method when less than 50% of the items were missing for the SDQ hyperactivity scale, the Carey temperament scores, and the Denver Score.

The Activity Subscale of the Carey Scale is a weighted sum of 9 component variables and prorated scores were calculated only when four components or less were missing. The Distractibility Score of the Carey Scale is a weighted sum of 10 component variables and prorated scores were calculated only when five components or less were missing.

There are 16 fine motor and 12 gross motor items in the ALSPAC adapted version of Denver Developmental Test. When three or fewer variables were missing we used the prorated scores. The prorated score assigns the mean of that child’s available scores to any missing variables.

A similar method was applied with the 5 items of the Hyperactivity/inattention subscale of the SDQ Questionnaire.

Table S1. Correlation Matrix

|  | 1 | 2 | 3 | 4 | 5 | 6 | 7 | 8 | 9 | 10 | 11 | 12 | 13 | 14 | 15 | 16 | 17 | 18 | 19 |
| --- | --- | --- | --- | --- | --- | --- | --- | --- | --- | --- | --- | --- | --- | --- | --- | --- | --- | --- | --- |
| SDQ Hyperactivity | .17** | .097** | .048** | .011 | .025 | .031* | .032* | .031* | .016 | .266** | .039** | .135** | .073** | .123** | .010 | .011 | .022 | .036** | .097** |
| DAWBA ADHD | .09* | .035** | .033** | -.009 | .007 | .032* | .018 | -.012 | .033** | .074** | -.010 | .069** | .051** | .039** | .023 | .011 | .012 | .002** | .043** |
| Mother <20 years | .001 | .093** | 1 | .025* | .014 | -.017 | .003 | .027* | -.003 | .007 | -.031** | .010 | -.009 | .012 | -.030** | .022 | .034** | .011 | .062** |
| Father >45 years | .011 | .017 | .025* | 1 | .003 | -.016 | .016 | -.010 | -.012 | .002 | .019 | -.014 | -.026* | -.025* | -.018 | .021 | .000 | .007 | -.007 |
| APGAR<10 | .001 | .014 |  |  | 1 | .084** | .034* | .045** | .013 | .028 | .012 | .020 | -.027 | .031* | .022 | -.023 | -.009 | .002 | -.013 |
| Prematurity | .033* | .010 |  |  |  | 1 | .080** | .477** | .085** | -.009 | .000 | .071** | .063** | .093** | .089** | .001 | .010 | -.005 | .015 |
| IUGR | -.013 | .006 |  |  |  |  | 1 | .203** | .001 | .016 | .003 | .008 | -.003 | .004 | -.002 | .009 | .006 | .025 | -.006 |
| HC<-2SD | .005 | .014 |  |  |  |  |  | 1 | .008 | .004 | .014 | .049** | .001 | .065** | .029* | .005 | .025 | .010 | .014 |
| Eye/Hearing centre | .012 | .017 |  |  |  |  |  |  | 1 | -.006 | -.011 | .011 | -.008 | .031** | .031** | -.003 | -.003 | -.013 | -.007 |
| Activity | .084** | .020 |  |  |  |  |  |  |  | 1 | .160** | .036** | .021 | .020 | -.075** | -.002 | .014 | .042** | .063** |
| Distractibility | -.026* | -.011 |  |  |  |  |  |  |  |  | 1 | -.031** | -.020 | -0.09 | -.014 | -.003 | .017 | .018 | -.002 |
| Vocabulary | .154** | .087** |  |  |  |  |  |  |  |  |  | 1 | .474** | .164** | .140** | .005 | -.002 | .015 | .018 |
| Grammar | .109** | .024* |  |  |  |  |  |  |  |  |  |  | 1 | .093** | .107** | -.002 | .013 | .002 | .012 |
| Fine motor | .042** | .065** |  |  |  |  |  |  |  |  |  |  |  | 1 | .157** | -.005 | .017 | .019 | .016 |
| Gross motor | -.026* | -.045** |  |  |  |  |  |  |  |  |  |  |  |  | 1 | .001 | .013 | .001 | -.022 |
| Sleeping difficulties | -.003 | .011 |  |  |  |  |  |  |  |  |  |  |  |  |  | 1 | .077** | .122** | -.016 |
| Crying difficulties | -.011 | .006 |  |  |  |  |  |  |  |  |  |  |  |  |  |  | 1 | .068** | .000 |
| Feeding difficulties | .013 | .015 |  |  |  |  |  |  |  |  |  |  |  |  |  |  |  | 1 | -.013 |
| Z-score ADHD PRS | -.002 | .069** |  |  |  |  |  |  |  |  |  |  |  |  |  |  |  |  | 1 |

1: Male: sex, 2: SES, 3: Maternal age at birth below 20 years, 4: paternal age at birth below 45 years, 5: APGAR<10, 6: Prematurity, 7: IUGR, 8: Head circumference <-2SD, 9: Referral to eye or hearing centre, 10: Carey Temperament Activity Score, 11: Carey Temperament Distractibility Score, 12: McArthur Vocabulary score <-1 SD, 13: McArthur Grammar score <-1SD, 14: Denver Fine Motor score <-1 SD, 15: Denver Gross Motor Score <-1 SD, 16: sleeping difficulties score >1 SD, 17: crying difficulties score >1SD, 18: feeding difficulties score >1SD, 19: z-score ADHD PRS

No multicollinearity: r coefficients < 0.9

*p<0.05 **p<0.01

| Table S2. Associations between ADHD Symptoms and early markers stratified by sex | | | | | | | | | | | | | | | |
| --- | --- | --- | --- | --- | --- | --- | --- | --- | --- | --- | --- | --- | --- | --- | --- |
| Predictors | Model 1 | | | | | | Model 2 (adjusted by SES) | | | | | Multivariable (adjusted SES)  Male: R2:0.094, Female: 0.091 | | | |
|  | Sex | N | Unstandard. B (95%CI) | Stand. B | p | N | | Unstandard. B (95%CI) | Stand. B | P | N | | Unstandard. B (95%CI) | Stand. B | p |
| Child ADHD PRS 0.05 | Males | 2815 | 0.270  (0.179, 0.360) | 0.109 | <0.001 | 2414 | | 0.233  (0.136, 0.331) | 0.095 | <0.001 | 1244 | | 0.206  (0.073, 0.339) | 0.083 | 0.002 |
|  | Females | 2694 | 0.189  (0.108, 0.270) | 0.088 | <0.001 | 2305 | | 0.161  (0.073, 0.249) | 0.075 | <0.001 | 1886 | | 0.171  (0.077, 0.265) | 0.079 | <0.001 |
| Mother <20 years at birth | Males | 4081 | 0.660  (0.244, 1.07) | 0.049 | 0.002 | 3428 | | 0.405  (-0.100, 0.910) | 0.027 | 0.116 | - | | - | - | - |
|  | Females | 3895 | 0.604  (0.227, 0.982) | 0.050 | 0.002 | 3275 | | 0.387  (-0.075, 0.850) | 0.029 | 0.101 | - | | - | - | - |
| Father >45 year at birth | Males | 3579 | 0.481  (-0.108, 1.07) | 0.027 | 0.110 | 3111 | | 0.332  (-0.289, 0.952) | 0.019 | 0.295 | - | | - | - | - |
|  | Females | 3375 | -0.164  (-0.660, 0.332) | -0.011 | 0.518 | 2917 | | 0.145  (-0.677, 0.387) | -0.010 | 0.593 | - | | - | - | - |
| Prematurity | Males | 2590 | 0.173  (-0.18, 0.53) | 0.019 | 0.338 | 2163 | | 0.199  (-0.188, 0.585) | 0.022 | 0.313 | - | | - | - | - |
|  | Females | 2457 | 0.336  (-0.031, 0.703) | 0.036 | 0.073 | 2067 | | 0.308  (-0.113, 0.728) | 0.032 | 0.151 | - | | - | - | - |
| I.U.G.R. | Males | 2609 | 0.815  (0.215, 1.41) | 0.052 | 0.008 | 2179 | | 0.704  (0.061, 1.348) | 0.046 | 0.032 | 1244 | | 0.272  (-0.530, 1.07) | 0.018 | 0.506 |
|  | Females | 2473 | 0.191  (-0.335, 0.717) | 0.014 | 0.476 | 2081 | | 0.442  (-0.137, 1.020) | 0.033 | 0.134 | - | | - | - | - |
| HC<-2SD at birth | Males | 2767 | 0.380  (-0.228, 0.987) | 0.023 | 0.220 | 2311 | | 0.367  (-0.313, 1.046) | 0.022 | 0.313 | - | | - | - | - |
|  | Females | 2636 | 0.614  (0.051, 1.176) | 0.042 | 0.033 | 2187 | | 0.466  (-0.18, 1.11) | 0.030 | 0.157 | - | | - | - | - |
| APGAR<10 | Males | 2502 | 0.084  (-0.113, 0.282) | 0.017 | 0.401 | 2083 | | 0.019  (-0.195, 0.233) | 0.004 | 0.865 | - | | - | - | - |
|  | Females | 2372 | 0.153  (-0.028, 0.334) | 0.092 | 0.097 | 1994 | | 0.177  (-0.020, 0.373) | 0.039 | 0.078 | - | | - | - | - |
| Hear/Vision referral | Males | 4024 | 0.106  (-0.237, 0.448) | 0.010 | 0.546 | 3398 | | 0.165  (-0.209, 0.538) | 0.015 | 0.388 | - | | - | - | - |
|  | Females | 3830 | 0.214  (-0.12, 0.549) | 0.020 | 0.208 | 3211 | | 0.150  (-0.223, 0.523) | 0.014 | 0.430 | - | | - | - | - |
| Fine motor  <-1SD | Males | 3978 | 0.838  (0.633, 1.044) | 0.126 | <0.001 | 3350 | | 0.785  (0.564, 1.006) | 0.119 | <0.001 | 1244 | | 0.906  (0.534, 1.279) | 0.130 | <0.001 |
|  | Females | 3742 | 0.690  (0.481, 0.898) | 0.106 | <0.001 | 3150 | | 0.638  (0.406, 0.870) | 0.096 | <0.001 | 1886 | | 0.362  (0.069, 0.656) | 0.054 | 0.016 |
| Gross motor  <-1SD | Males | 3988 | 0.071  (-0.17, 0.318) | 0.009 | 0.574 | 3357 | | 0.058  (-0.206, 0.322) | 0.007 | 0.666 | - | | - | - | - |
|  | Females | 3767 | 0.139  (-0.074, 0.351) | 0.021 | 0.202 | 3170 | | 0.165  (-0.066, 0.397) | 0.025 | 0.162 | - | | - | - | - |
| Vocabulary  <-1SD | Males | 4017 | 0.726  (0.549, 0.902) | 0.126 | <0.001 | 3374 | | 0.575  (0.380, 0.769) | 0.099 | <0.001 | 1244 | | 0.490  (0.081, 0.898) | 0.072 | 0.019 |
|  | Females | 3815 | 0.592  (0.376, 0.809) | 0.087 | <0.001 | 3202 | | 0.623  (0.379, 0.867) | 0.088 | <0.001 | 1886 | | 0.287  (-0.119, 0.694) | 0.035 | 0.166 |
| Grammar  <-1SD | Males | 3643 | 0.390  (0.191, 0.589) | 0.063 | <0.001 | 3065 | | 0.293  (0.08, 0.506) | 0.048 | 0.007 | 1244 | | 0.062  (-0.317, 0.440) | 0.010 | 0.750 |
|  | Females | 3663 | 0.299  (0.076, 0.523) | 0.043 | 0.009 | 3083 | | 0.410  (0.165, 0.656) | 0.059 | 0.001 | 1886 | | 0.048  (-0.297, 0.393) | 0.007 | 0.784 |
| Activity  Carey | Males | 3999 | 0.141  (0.124, 0.157) | 0.258 | <0.001 | 3362 | | 0.141  (0.123, 0.158) | 0.261 | <0.001 | 1244 | | 0.113  (0.084, 0.142) | 0.209 | <0.001 |
|  | Females | 3792 | 0.121  (0.107, 0.136) | 0.253 | <0.001 | 3187 | | 0.127  (0.111, 0.143) | 0.263 | <0.001 | 1886 | | 0.125  (0.104, 0.146) | 0.262 | <0.001 |
| Distractibility Carey | Males | 3998 | 0.025  (0.009, 0.041) | 0.048 | 0.003 | 3362 | | 0.031  (0.014, 0.048) | 0.060 | <0.001 | 1244 | | 0.009  (-0.019, 0.037) | 0.018 | 0.516 |
|  | Females | 3792 | 0.019  (0.004, 0.034) | 0.041 | 0.012 | 3187 | | 0.023  (0.007, 0.04) | 0.050 | 0.005 | 1886 | | 0.019  (-0.002, 0.039) | 0.040 | 0.074 |
| Sleeping  >1SD | Males | 3957 | 0.007  (-0.215, 0.230) | 0.011 | 0.949 | 3223 | | 0.028  (-0.211, 0.266) | 0.004 | 0.820 | - | | - | - | - |
|  | Females | 3669 | 0.154  (-0.049, 0.357) | 0.025 | 0.137 | 3053 | | 0.127  (-0.096, 0.350) | 0.020 | 0.266 | - | | - | - | - |
| Crying  >1SD | Males | 3889 | 0.091  (-0.224, 0.406) | 0.009 | 0.571 | 3250 | | 0.065  (-0.272, 0.403) | 0.007 | 0.705 | - | | - | - | - |
|  | Females | 3703 | 0.362 (  0.083, 0.640) | 0.042 | 0.011 | 3077 | | 0.196  (-0.120, 0.512) | 0.022 | 0.224 | - | | - | - | - |
| Feeding  >1SD | Males | 3933 | 0.107  (-0.083, 0.297) | 0.018 | 0.271 | 3290 | | 0.150  (-0.056, 0.356) | 0.025 | 0.153 | - | | - | - | - |
|  | Females | 3735 | 0.298  (0.119, 0.477) | 0.053 | 0.001 | 3100 | | 0.269  (0.072, 0.465) | 0.048 | 0.007 | 1886 | | 0.150  (-0.09, 0.390) | 0.027 | 0.220 |

ADHD: Attention Deficit and Hyperactivity Disorder, N: total number, Unstandard. B: Unstandardized Beta, Stand. B: Standardized Beta, p: p value. PRS: Polygenic Risk Scores, IUGR: Intrauterine Growth Restriction, HC: head circumference, SD: standard deviation

| Table S3. Associations between ADHD diagnosis and early markers in Males | | | | | | | | | | | | |
| --- | --- | --- | --- | --- | --- | --- | --- | --- | --- | --- | --- | --- |
| Predictors | Model 1 | | | | Model 2 (adjusted by SES) | | | | Multivariable  N=1942 | | | |
|  | N | OR (95%CI) | B (SE) | p | N | OR (95% CI) | B (SE) | p | N | OR (95% CI) | B (SE) | p |
| ADHD PRS | 2810 | 1.32  (1.08, 1.61) | 0.279  (0.101) | 0.006 | 2389 | 1.33  (1.07, 1.66) | 0.288  (0.113) | 0.010 | 1942 | 1.27  (0.97, 1.65) | 0.24  (0.13) | 0.077 |
| Mother <20 years at birth | 3942 | 1.85  (0.89, 3.87) | 0.618  (0.376) | 0.100 | 3312 | 1.08  (0.391, 3.028) | 0.085  (0.522) | 0.871 | - | - | - | - |
| Father >45 year at birth | 3462 | 0.55  (0.19, 1.55) | -0.586  (0.524) | 0.263 | 3017 | 0.46  (0.16, 1.30) | -0.771  (0.529) | 0.145 | - | - | - | - |
| Prematurity | 2500 | 1.35  (0.71, 2.58) | 0.307  (0.327) | 0.349 | 2090 | 1.48  (0.75, 2.92) | 0.392  (0.347) | 0.258 | - | - | - | - |
| I.U.G.R. | 2522 | 2.27  (0.96, 5.39) | 0.823  (0.440) | 0.061 | 2108 | 2.12  (0.82, 5.45) | 0.751  (0.482) | 0.119 | - | - | - | - |
| HC<-2SD at birth | 2689 | 0.449  (0.06, 3.27) | -0.800  (1.014) | 0.430 | 2238 | 0.57  (0.079, 4.24) | -0.549  (1.02) | 0.589 | - | - | - | - |
| APGAR<10 | 2414 | 0.96  (0.64, 1.44) | -0.037  (0.205) | 0.857 | 2012 | 1.02  (0.66, 1.58) | 0.021  (0.224) | 0.926 | - | - | - | - |
| Hear/Vision referral | 3898 | 2.00  (1.08, 3.68) | 0.693  (0.312) | 0.026 | 3290 | 2.14  (1.09, 4.19) | 0.759  (0.344) | 0.027 | 1942 | 2.16  (0.82, 5.67) | 0.77  (0.49) | 0.119 |
| Fine motor  <-1SD | 3816 | 1.69  (1.11, 2.58) | 0.529  (0.214) | 0.014 | 3217 | 1.72  (1.09, 2.72) | 0.544  (0.234) | 0.020 | 1942 | 1.41  (0.74, 2.69) | (0.80, 2.40) | 0.302 |
| Gross motor  <-1SD | 3823 | 1.38  (0.83, 2.30) | 0.325  (0.26) | 0.211 | 3222 | 1.50  (0.87, 2.59) | 0.408  (0.28) | 0.143 | - | - | - | - |
| Vocabulary  <-1SD | 3856 | 1.85  (1.28, 2.66) | 0.615  (0.186) | 0.001 | 3240 | 1.77  (1.18, 2.65) | 0.57  (0.20) | 0.006 | 1942 | 1.54  (0.77, 3.06) | 0.431  (0.35) | 0.219 |
| Grammar  <-1SD | 3504 | 1.70  (1.13, 2.57) | 0.53  (0.21) | 0.011 | 2948 | 1.62  (1.03, 2.56) | 0.48  (0.23) | 0.037 | 1942 | 1.54  (0.80, 2.98) | 0.435  (0.335) | 0.195 |
| Activity  Carey | 3835 | 1.12  (1.08, 1.17) | 0.115  (0.02) | <0.001 | 3229 | 1.13  (1.08, 1.18) | 0.126  (0.02) | <0.001 | 1942 | 1.13  (1.06, 1.19) | 0.12  (0.03) | <0.001 |
| Distractibility Carey | 3833 | 0.98  (0.95, 1.03) | -0.012  (0.019) | 0.536 | 3229 | 0.97  (0.94, 1.02) | -0.023  (0.021) | 0.260 | - | - | - | - |
| Sleeping  >1SD | 3756 | 1.16  (0.72, 1.88) | 0.154  (0.24) | 0.526 | 3120 | 1.09  (0.63, 1.88) | 0.092  (0.277) | 0.739 | - | - | - | - |
| Crying  >1SD | 3785 | 0.963  (0.46, 1.99) | -0.038  (0.371) | 0.919 | 3146 | 0.97  (0.42, 2.24) | -0.030  (0.428) | 0.943 | - | - | - | - |
| Feeding  >1SD | 3829 | 0.96  (0.61, 1.49) | -0.041  (0.23) | 0.857 | 3186 | 0.69  (0.40, 1.18) | -0.367  (0.276) | 0.183 | - | - | - | - |

ADHD: Attention Deficit and Hyperactivity Disorder, N: total number, Unstandard. B: Unstandardized Beta, Stand. B: Standardized Beta, p: p value. PRS: Polygenic Risk Scores, IUGR: Intrauterine Growth Restriction, HC: head circumference, SD: standard deviation

Table S4. Comparison of outcome variables between those with complete precursor data to those with any missing data.

|  | | Complete predictors data  N=2108 (22.9%) | Partial Predictors data  N=7093 (77.1%) | T test/Chi square |
| --- | --- | --- | --- | --- |
| Outcome variables | |  | | |
| DAWBA DSM IV ADHD diagnosis | N | 38 | 136 | χ2(1)=0.523  p=0.470 |
| No DAWBA DSM IV ADHD diagnosis | N | 1924 | 6023 |  |
| SDQ Hyperactivity Score >= 7 | Mean (SD) | 8.68 (0.825) | 8.75 (0.797) | t(528)=0.907,  p=0.365 |

Figure S1. Histogram of missing predictors


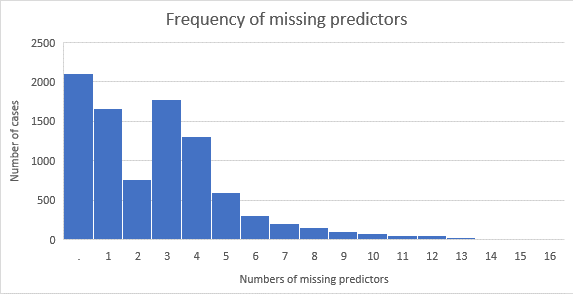


**References**

Boyd A, Golding J, Macleod J, Lawlor DA, Fraser A, Henderson J *et al.* Cohort Profile: the 'children of the 90s'--the index offspring of the Avon Longitudinal Study of Parents and Children. *International journal of epidemiology* 2013; 42(1): 111-127.

Euesden J, Lewis CM, O'Reilly PF. PRSice: Polygenic Risk Score software. Bioinformatics 2015; 31(9): 1466-1468.

Fraser A, Macdonald-Wallis C, Tilling K, Boyd A, Golding J, Davey Smith G *et al.* Cohort Profile: the Avon Longitudinal Study of Parents and Children: ALSPAC mothers cohort. *International journal of epidemiology* 2013; 42(1): 97-110.

Northstone K, Lewcock M, Groom A, Boyd A, Macleod J, Timpson N *et al.* The Avon Longitudinal Study of Parents and Children (ALSPAC): an update on the enrolled sample of index children in 2019. *Wellcome Open Res* 2019; 4: 51-51.

Schizophrenia Working Group of the Psychiatric Genomics Consortium. Biological insights from 108 schizophrenia-associated genetic loci. Nature 2014; 511(7510): 421-427.

Winsper, C., & Wolke, D. (2014). Infant and toddler crying, sleeping and feeding problems and trajectories of dysregulated behavior across childhood. Journal of Abnormal Child Psychology, 42(5), 831–843.
